# Virulence and Vertically Transmitted Pathogens: The Role of Costs Paid by Co‐Evolving Hosts in a Self‐Regulating Population

**DOI:** 10.1002/ece3.71840

**Published:** 2025-07-31

**Authors:** Bita Ghodsi, Geoff Wild

**Affiliations:** ^1^ Department of Mathematics Western University London Ontario Canada

**Keywords:** adaptive dynamics, evolutionary epidemiology, infectious disease, mathematical model

## Abstract

Many pathogens transmit horizontally through usual routes and vertically from parent to offspring. Co‐evolution is predicted, under certain circumstances, to produce a positive relationship between host‐pathogen antagonism and the rate of vertical transmission. We cannot disentangle the roles of host demographics and the costs of host immune function in establishing this pattern. On one hand, models that assume no density‐dependent growth of host populations and limit the cost of immune function to infected hosts only predict that the positive relationship is possible. On the other hand, models that assume density‐dependent growth of host populations and impose the cost of immune function on all hosts, regardless of infection status, suggest that the positive relationship is not possible. Here, we seek to resolve the confusion. We model the co‐evolution of a host and its pathogen when the latter can transmit both vertically and horizontally. We assume host population growth is self‐limiting, and we impose the cost of immune function only on infected hosts. We find that a positive relationship between host‐pathogen antagonism and vertical transmission is possible under our assumptions. Our finding points to the critical role played by assumptions about when hosts pay the cost of immune function. We also find that the combination of density‐dependent host population growth and the cost‐free lifestyle of uninfected hosts raises the possibility of selection‐driven pathogen extinction. We discuss our findings in relation to previous theory and empirical findings.

## Graphical abstract

Theory suggests that coevolution can lead to a positive relationship between host‐pathogen antagonism and vertical transmission, but it’s uncertain whether host demographics or the nature of immune costs drive this pattern. We address this uncertainty with a mathematical model of host‐pathogen co‐evolution that allows for both vertical and horizontal transmission of the pathogen. We find that self‐regulation of the host does not always lead to lower virulence of vertically transmitted pathogens.

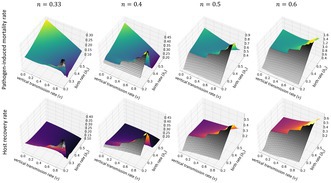

## 1 Introduction

Pathogens can be transmitted vertically from parent to offspring or horizontally through non‐hereditary means. Humans and other mammals (Montoya and Liesenfeld 2004; Arora et al. 2017), insects (Werren 1997), and plants (Pagán 2022) are all affected by pathogens that can transmit vertically.

Vertical transmission is predicted to select against pathogens that inflict excessive harm on their hosts, that is pathogens that are too virulent (Ewald 1994; Frank 1996; Lipsitch et al. 1996; Stearns and Medzhitov 2015; Cressler et al. 2016; Úbeda and Jansen 2016). However, virulence generally reflects traits expressed by the host as well (Read 1994; Day 2002; Day and Burns 2003). In fact, harmful disease outcomes often result from the host's response to infection and how that response interacts with the pathogen (Clark et al. 2004; Griffin 2010; Fajgenbaum and June 2020). Given that vertical transmission is also predicted to select for changes in a host's immune response (Mitchell et al. 2022), it is difficult to forecast how co‐evolutionary processes will shape disease outcomes when the vertical transmission route is available to pathogens.

The contrasting results of recent modeling efforts emphasize how difficult it is to forecast patterns in host‐pathogen co‐evolution when vertical transmission is possible. One set of results suggests a higher vertical transmission rate can easily produce more severe disease outcomes over evolutionary time (Shillcock et al. 2023), while another set suggests that higher vertical transmission never leads to more severe disease outcomes (Brotman and Wild 2025). The discrepancy may stem from the different modeling assumptions used. Shillcock et al. (2023) base their predictions on a demographic model in which pathogen‐induced mortality is the sole factor regulating host‐population growth. They also assume hosts pay the cost of immune function only when infected by the pathogen. By contrast, Brotman and Wild (2025) limit the growth of the host population with both pathogen‐induced mortality and a negative density‐dependent host birth rate. In addition, Brotman and Wild (2025) assume hosts pay the cost of immune function regardless of their infection status. This raises the question: Is negative density‐dependent growth of the host population the key to ensuring a tight link between vertical transmission and the evolution of reduced pathogen virulence? It also leads us to ask whether the link between vertical transmission and reduced pathogen virulence can be broken if we release uninfected hosts from the cost of immune function.

We address these questions using an adaptive dynamics model of co‐evolution between a vertically transmitted pathogen and its host. Our model imagines that both host and pathogen are haploid and asexual, like *Escherichia coli* and bacteriophage systems (Levin and Lenski 1985) or protozoa and their bacterial parasites (Kaltz and Koella 2003). We assume host population growth is self‐regulated, and hosts pay the cost of immune function only when infected. Although we aim to distinguish our work from that of previous authors, we follow previous authors in determining how host demography and the ease with which infections are created horizontally (later referred to as the ‘profitability of horizontal transmission’) interact with vertical transmission to shape co‐evolutionary outcomes. Unlike previous authors, we find that the selection can drive vertically transmitted pathogens to very low densities when they exploit a self‐regulating host population.

## 2 Materials and Methods

### 2.1 Ecological Model

We adopt a standard model for the dynamics of a host population in the presence of an infectious agent. We use $S=S\left(t\right)$ to represent the number of host individuals at time t who are uninfected but susceptible to infection in the future. We use $E=E\left(t\right)$ to represent the number of host individuals infected by the pathogen but not yet infectious (they are ‘exposed’ only). Finally, we use $I=I\left(t\right)$ to denote the number of infected host individuals who are also infectious. Our immediate aim is to describe the changes in S, E, and I over time and to predict the long‐term dynamic behavior of the system.

The number of individuals of each category varies due to death and birth. We use $b_{s}$ to denote the per‐capita birth rate of susceptible and exposed hosts, respectively, and $b_{I}$ to denote the per‐capita birth rate of infectious hosts. We use $\mu \left(N\right)=\mu_{0}N$ to denote the per‐capita background mortality rate of hosts, where $\mu_{0}$ is a positive constant and N=S+E+I is the total population size. We assume the infection increases the per‐capita mortality rate of infectious (but not exposed) individuals by α, and we refer to α as the pathogen‐induced mortality rate.

The number of individuals in each category also varies due to disease progression, transmission, and recovery. First, we assume that exposed hosts progress to become infectious at a per‐capita rate δ. Second, we assume infectious hosts transmit the pathogen horizontally to susceptible individuals at rate βS, where β is a positive constant that characterizes the pathogen's horizontal transmissibility. In addition, we assume infectious hosts transmit the pathogen vertically to their newborn offspring with probability v. Newborns who acquire an infection from their parent join the exposed class (all other newborns join the susceptible class). Finally, we assume infectious hosts recover from an infection at a per‐capita rate γ, and that recovered hosts are immediately susceptible.

For later use, we introduce a trade‐off between the pathogen‐induced mortality rate, α, and the horizontal transmission rate constant, β. We capture the trade‐off by treating β as an increasing function of α. Specifically, we assume $\beta =\beta \left(\alpha \right)=k\alpha^{n}$, where k is a positive scaling constant and 0<n<1 represents the profitability of the horizontal transmission. The idea that underlies this trade‐off is that greater transmissibility (larger β) inflicts greater harm on the host (larger α). Here, a one‐percent increase in pathogen‐induced mortality rate yields an n‐percent increase in the horizontal transmission rate. Relationships between harm and horizontal transmission, like the one we assume, are supported by data (Acevedo et al. 2019). Though the relationships have been controversial, they are recognized as critical for accurately understanding pathogen evolution (Alizon et al. 2009).

We also introduce a trade‐off between the host recovery rate, γ, and host birth rate while infectious, $b_{I}$. Specifically, we assume $b_{I}=b_{I}\left(\gamma \right)=b_{s}-c\gamma^{2}$, where c is a scaling constant that is small enough to ensure $b_{I}$ remains positive. We restrict ourselves to a quadratic relationship because it approximates a Gaussian one (Shillcock et al. 2023). The underlying idea is that the host must allocate resources to immune‐system function (larger γ), which implies fewer resources are available for reproduction (smaller $b_{I}$). Evidence from insects aligns with the trade‐off we assume here, as it indicates that increased resistance to ectoparasites reduces host fecundity (Luong and Polak 2007). Indirect evidence for binding resource constraints might also come from studies of sexual selection, where individuals advertise good health with costly coloration or ornaments (Hamilton and Zuk 1982).

We have not imposed the cost of immune function on exposed hosts. We made this assumption because our model does not allow exposed hosts to recover, transmit horizontally or vertically, or die from their infection; ultimately, this simplification makes our model more straightforward at the cost of some realism. While assumptions about when hosts are faced with the costs of recovery certainly matter (Shillcock et al. 2023; Brotman and Wild 2025), the key consideration is whether all (or not all) hosts pay said cost. Imposing the cost on exposed hosts would not change the fact that our model still allows some hosts (namely, susceptibles) to live cost‐free.

We can summarize the ecological dynamics described above as

(1) $$\begin{array}{l} \frac{\mathrm{dS}}{\mathrm{dt}}=b_{s}S+b_{s}E+\left(1-v\right)b_{I}\left(\gamma \right)I-\beta \left(\alpha \right)\text{IS}+\mathrm{\gamma I}-\mu \left(N\right)S,\\ \frac{\mathrm{dE}}{\mathrm{dt}}=\beta \left(\alpha \right)\text{IS}+{\mathrm{vb}}_{I}\left(\gamma \right)I-\mathrm{\delta E}-\mu \left(N\right)E,\\ \frac{\mathrm{dI}}{\mathrm{dt}}=\mathrm{\delta E}-\mathrm{\gamma I}-\mathrm{\alpha I}-\mu \left(N\right)I. \end{array}$$

As we shall see, the exposed hosts play only a minor background role in the evolutionary dynamics of this system.

The dynamics captured by Equation (1) can support an equilibrium state where the pathogen is absent and the size of the host population is ${\bar{N}}_{0}=\frac{b_{s}}{\mu_{0}}$. We can use a linear stability analysis to show that this ‘disease‐free equilibrium’ is unstable whenever

(2) $$\frac{\delta }{\delta +\mu \left({\bar{N}}_{0}\right)}\frac{\beta \left(\alpha \right){\bar{N}}_{0}+{\mathrm{vb}}_{I}\left(\gamma \right)}{\gamma +\alpha +\mu \left({\bar{N}}_{0}\right)}>1.$$

**FIGURE 1:**
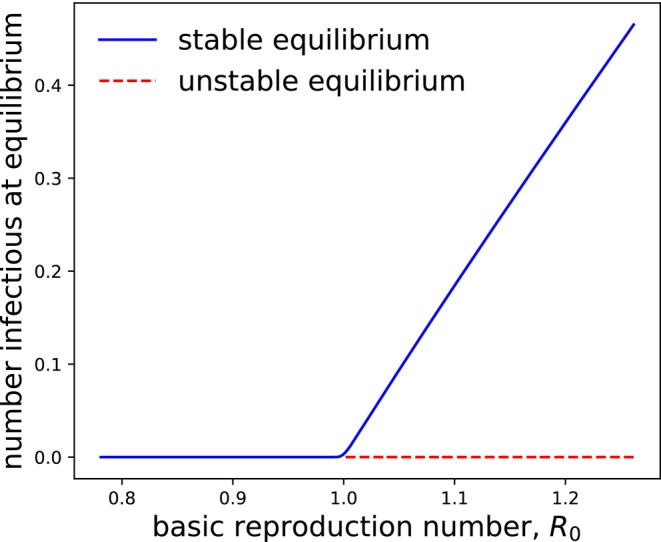
Long‐run number of infectious hosts $I\left(\infty \right)$, plotted as a function of the basic reproduction number, $R_{0}$ (left‐hand side of Condition 2). As the basic reproduction number passes through a value of 1, the disease‐free equilibrium, where $I\left(\infty \right)=0$, becomes unstable and its stability is transferred to an endemic equilibrium, where $I\left(\infty \right)>0$. Numerical experiments shown here are based on varying α from 3.0 to 7.0, with $b_{s}=1.5$, $b_{I}\left(\gamma \right)=1.0$, $\mu_{0}=0.15$, $\beta \left(\alpha \right)=1.2$, v=0.3, γ=2.0, δ=3.0.

The left‐hand side of Condition (2) is known as the basic reproduction number, often denoted $R_{0}$. It gives the expected number of new horizontal and vertical infections generated by an exposed host in an otherwise fully susceptible population (van den Driessche and Watmough 2002). When the basic reproduction number is greater than 1, the index case does better than simply replacing itself, so the disease‐free equilibrium loses its stability. Moreover, when the basic reproduction number is greater than 1, we expect a new equilibrium to emerge, where the pathogen is endemic. Numerical experiments show that when Condition (2) holds, the disease‐free equilibrium for Equation (1) is unstable and the host population tends to an endemic equilibrium where positive numbers of infectious individuals circulate (Figure 1).

### 2.2 Co‐Evolutionary Model

To model the co‐evolution of pathogen and host, we must determine the fitness of each. Our models for fitness track the fate of rare lineages of pathogens and hosts, respectively, when they are introduced to a resident host population near its endemic equilibrium, $S=\bar{S}$, $E=\bar{E}$, $I=\bar{I}$. The same general approach often appears in the literature (Abrams et al. 1993; Day and Burns 2003).

In Appendix A, we apply the approach of Hurford et al. (2010) to find the fitness of a mutant pathogen that increases the host mortality rate by $\alpha_{m}$, rather than by α. Provided the mutant pathogen is rare and the host population is near its endemic equilibrium, the mutant pathogen's fitness is given by

(3) $$W_{p}\left(\alpha_{m};\alpha ,\gamma \right)=\frac{\delta }{\delta +\mu \left(\bar{N}\right)}\frac{\beta \left(\alpha_{m}\right)\bar{S}+{\mathrm{vb}}_{I}\left(\gamma \right)}{\gamma +\alpha_{m}+\mu \left(\bar{N}\right)},$$

where $\bar{N}$ is the size of the host population at the endemic equilibrium. We note that both $\bar{S}$ and $\bar{N}$ are functions of α and γ. The mutant pathogen invades if $W_{p}\left(\alpha_{m};\alpha ,\gamma \right)>W_{p}\left(\alpha ;\alpha ,\gamma \right)=1$, and if the inequality is reversed, the mutant is eliminated. Provided selection is weak (and provided an additional, minor technical condition is met), a mutant pathogen that invades, eventually displaces the resident pathogen strain (Dercole and Rinaldi 2008).

In Appendix B, we apply the approach of Hurford et al. (2010) to find the fitness of a mutant host that recovers at rate $\gamma_{m}$, rather than γ. Mutant host fitness is expressed in terms of a ‘next‐generation matrix,’ $K_{h}$ (see Appendix B). The ijth entry of this matrix, $K_{h_{\mathrm{ij}}}$, gives the expected number of state i=S,E,I offspring produced by a host in state j=S,E,I throughout its lifetime. Provided the mutant host is rare and the resident system is near the endemic equilibrium, the mutant host's fitness is

(4) $$W_{h}\left(\gamma_{m};\alpha ,\gamma \right)=\frac{\left(K_{h_{11}}+K_{h_{22}}\right)+\sqrt{\left(K_{h_{11}}+K_{h_{22}}\right)-4\left(K_{h_{11}}K_{h_{22}}-K_{h_{21}}K_{h_{12}}\right)}}{2},$$

where, in general, $K_{h_{\mathrm{ij}}}$ depend on α and γ. The mutant host invades when $W_{h}\left(\gamma_{m};\alpha ,\gamma \right)$ is greater than 1 and is eliminated if $W_{h}\left(\gamma_{m};\alpha ,\gamma \right)$ is less than 1. Again, provided selection is weak, a mutant host that invades displaces the resident host strain (Dercole and Rinaldi 2008).

We model co‐evolution as a series of invasion‐displacement events: an advantageous mutant (either pathogen or host) arises and displaces the existing resident before another mutant (either pathogen or host) can arise (Metz et al. 1992). The rate of change of a trait expression over evolutionary time τ is proportional to the fitness gradient of the mutant traits evaluated at the resident trait value. This allows us to describe the trajectories of pathogen‐induced mortality and host recovery over evolutionary time τ as

(5) $$\begin{array}{l} \frac{\mathrm{d\alpha}}{\mathrm{d\tau}}=k_{p}\frac{\partial W_{p}}{\partial \alpha_{m}}\left(\alpha ;\alpha ,\gamma \right),\\ \frac{\mathrm{d\gamma}}{\mathrm{d\tau}}=k_{h}\frac{\partial W_{h}}{\partial \gamma_{m}}\left(\gamma ;\alpha ,\gamma \right), \end{array}$$

where $k_{p}$ and $k_{h}$ are constants that scale the rate of evolutionary change (Abrams et al. 1993; Dieckmann and Law 1996). The partial derivatives in Equation (5) indicate that the difference between the mutant and resident traits is only very small; in other words, selection is weak. Equation (5) implies that mutant hosts and mutant pathogens do not co‐occur. Thus, correlations between pathogen and host cannot emerge in this model.

A pair of host and pathogen traits, $\left(\alpha^{*},\gamma^{*}\right)$, are at co‐evolutionary equilibrium when they satisfy

(6) $$\begin{array}{l} \frac{\partial W_{p}}{\partial \alpha_{m}}\left(\alpha^{*};\alpha^{*},\gamma^{*}\right)=0\\ \frac{\partial W_{h}}{\partial \gamma_{m}}\left(\gamma^{*};\alpha^{*},\gamma^{*}\right)=0. \end{array}$$

In other words, the pair $\left(\alpha^{*},\gamma^{*}\right)$ is an equilibrium solution to Equation (5). That said, we consider a pair of traits at co‐evolutionary equilibrium to be convergence stable if they are asymptotically stable under the dynamics described by Equation (5).

In general, convergence stable pairs like $\left(\alpha^{*},\gamma^{*}\right)$ may still be susceptible to invasion by mutants (Geritz et al. 1998), that is, they may lack evolutionary stability (Maynard Smith 1982). To guarantee that $\left(\alpha^{*},\gamma^{*}\right)$ are evolutionarily stable, we would like to require

(7) $$\begin{array}{l} W_{p}\left(\alpha_{m};\alpha^{*},\gamma^{*}\right)<W_{p}\left(\alpha^{*};\alpha^{*},\gamma^{*}\right)=1\\ W_{h}\left(\gamma_{m};\alpha^{*},\gamma^{*}\right)<W_{h}\left(\gamma^{*};\alpha^{*},\gamma^{*}\right)=1 \end{array}$$

for all $\alpha_{m}\neq \alpha^{*}$ and $\gamma_{m}\neq \gamma^{*}$. However, we can only impose a local version of Condition (7); specifically, for $\alpha_{m}$ near $\alpha^{*}$ and $\gamma_{m}$ near $\gamma^{*}$, we have

(8) $$\begin{array}{l} \frac{\partial^{2}W_{p}}{\partial \alpha_{m}^{2}}\left(\alpha^{*};\alpha^{*},\gamma^{*}\right)<0,\\ \frac{\partial^{2}W_{h}}{\partial \gamma_{m}^{2}}\left(\gamma^{*};\alpha^{*},\gamma^{*}\right)<0. \end{array}$$

Thus, we use the previous pair of inequalities as our local condition for the evolutionary stability of $\left(\alpha^{*},\gamma^{*}\right)$.

### 2.3 Numerical Analysis

We identify convergence‐stable pairs numerically in four steps. First, we guess values for $\left(\alpha^{*},\gamma^{*}\right)$. Second, we find the endemic equilibrium corresponding to our guess by iterating Equation (1) forward in time. Third, we use the endemic equilibrium to calculate the fitness; specifically, we calculate finite difference approximations for the partial derivatives for Equation (5). Fourth, we update the guess by adding some multiple of the appropriate partial derivative to the current values of $\alpha^{*}$ and $\gamma^{*}$, respectively. We repeat steps two to four until the absolute values of the finite difference approximations to the fitness derivatives in Equation (5) become sufficiently close to zero. To ensure the pair $\left(\alpha^{*},\gamma^{*}\right)$ is also evolutionarily stable, we verify Condition (8) using finite difference approximations. We implemented the methodology in Python (Version 3.x) and have detailed each step above in the Jupyter notebook found in the Supplement.

## 3 Results

### 3.1 Preliminary Observations

**FIGURE 2:**
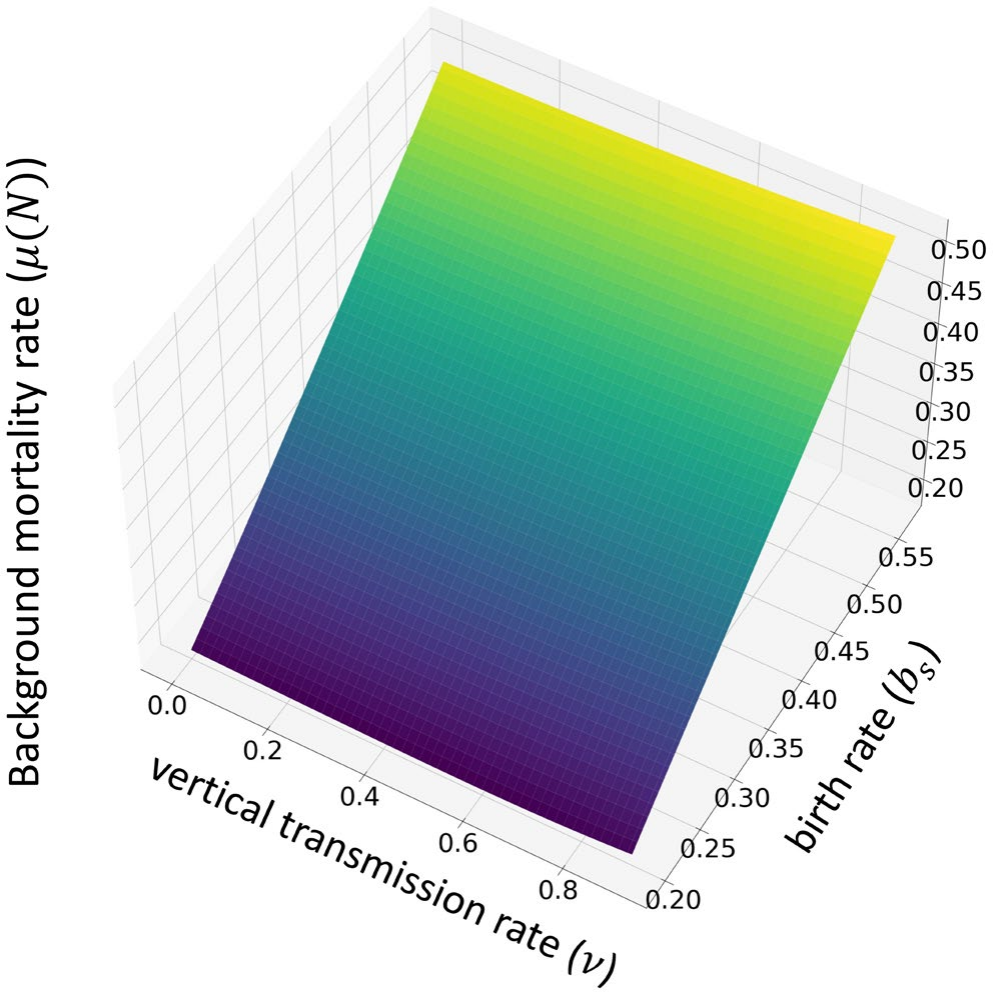
Variation of the background mortality rate when the population is at endemic and co‐evolutionary equilibrium. We assume the rate of exposed hosts becoming infectious, δ=1, horizontal transmission profitability, n=0.27, and the background mortality rate, $\mu \left(N\right)=0.15N$, where N represents the total population. In addition, k=1 and c=1.

Before we detail the results, it is useful to make three fundamental observations. First, an increase in the birth rate, $b_{s}$, prompts a compensatory rise in the background mortality rate, $\mu \left(\bar{N}\right)$, at equilibrium (e.g., Figure 2). Consequently, a rise in $b_{s}$ triggers a reduction in the expected duration of infection and the expected life span of the host at equilibrium. Second, as n increases, horizontal transmission becomes more profitable and, therefore, more attractive for the pathogen, meaning that there is a greater fitness incentive to invest in horizontal transmission. From the host's perspective, an increased n leads to a greater risk of pathogen‐induced mortality, as pathogen‐induced mortality is associated with horizontal transmission. Third, a higher vertical transmission rate, v, means a larger proportion of offspring from infected individuals will also be infected. From the host's perspective, infected offspring are costly because they are of lower quality; however, infected offspring are beneficial to the pathogen as the pathogen does not need to impose additional mortality on its host to create these new infections. These points facilitate a clearer understanding of the results.

The results themselves can be organized around the profitability of horizontal transmission, n. Generally, lower n leads to lower levels of trait expressions (i.e., smaller $\alpha^{*}$ and $\gamma^{*}$). By contrast, higher n leads to higher levels of trait expressions. However, within this overarching framework, additional patterns emerge. We detail these patterns below.

### 3.2 Lower Profitability of Horizontal Transmission

We make two sets of observations for cases in which the profitability of horizontal transmission, represented by n, is low. The first set of observations concerns the effect of model parameters on the stable pathogen‐induced mortality rate, $\alpha^{*}$. The second set of observations concerns the effect of model parameters on the stable host recovery rate, $\gamma^{*}$.

**FIGURE 3:**
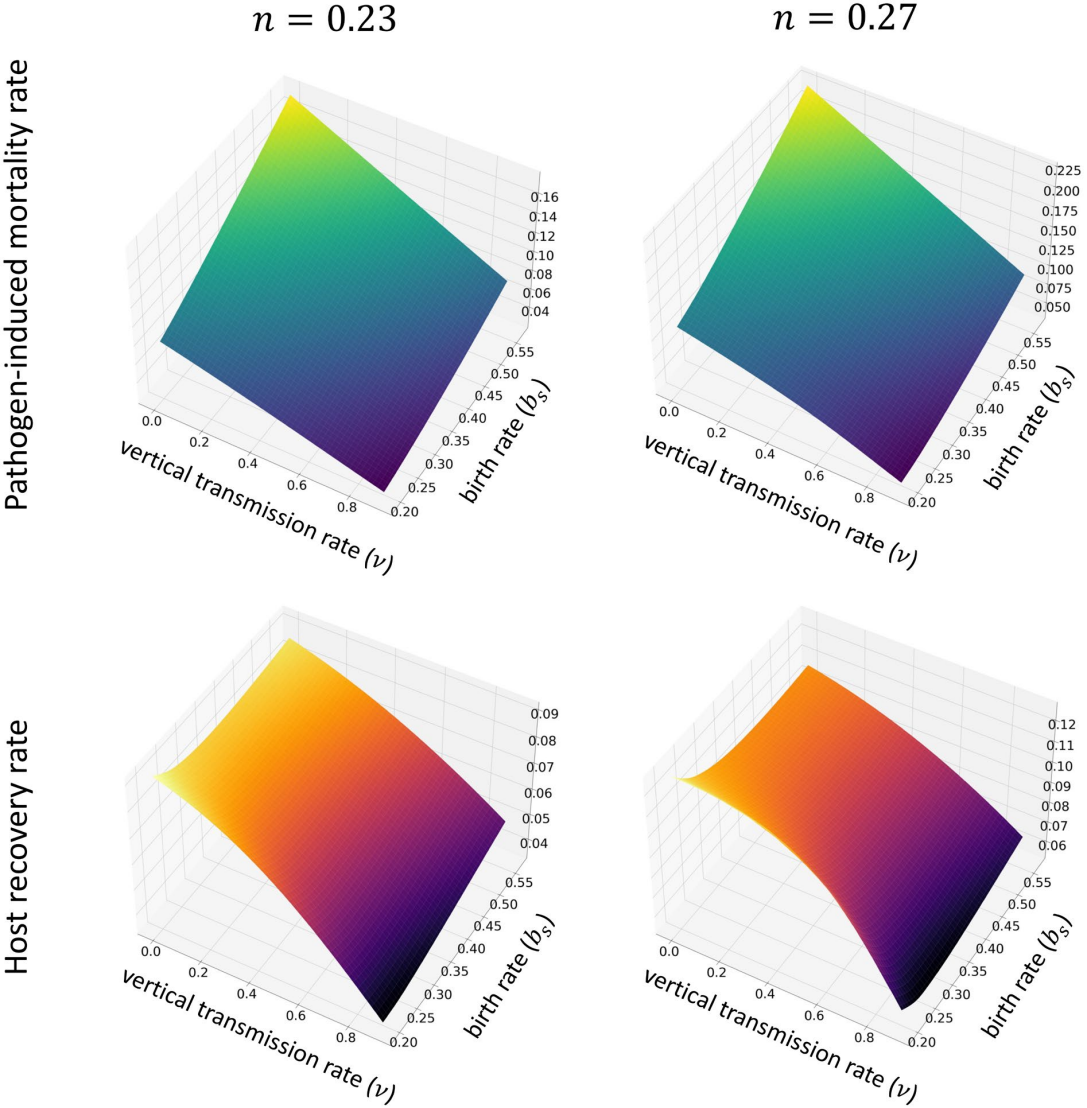
Variation in the evolutionarily stable values of pathogen‐induced mortality rate $\left(\alpha^{*}\right)$ and host recovery rate $\left(\gamma^{*}\right)$ across different levels of vertical transmission rates $\left(v\right)$ and birth rates $\left(b_{s}\right)$, at lower horizontal transmission profitability $\left(n\right)$, specifically for n=0.23 and n=0.27. The first row of subplots details how $\alpha^{*}$ adapts to shifts in vertical transmission and birth rates for these lower n values, while the second row focuses on $\gamma^{*}$. We assume the rate of exposed hosts becoming infectious, δ=1, and the background mortality rate, $\mu \left(N\right)=0.15N$, when N represents the total population. In addition, k=1 and c=1.

Modifying the vertical transmission, v, and birth rate, $b_{s}$, influences the value of $\alpha^{*}$. We observe that a rise in birth rate, $b_{s}$, elevates the pathogen‐induced mortality rate $\alpha^{*}$ (Figure 3). A higher birth rate results in higher background mortality, as explained above, and in turn shortens the expected duration of infection. To maintain its reproductive success, then, the pathogen must create new infections at a higher rate, and this need is reflected in the increased $\alpha^{*}$, which improves horizontal transmission. With $b_{s}$ held constant, an increase in v reduces the pathogen‐induced mortality rate, $\alpha^{*}$ (Figure 3). As opportunities for vertical transmission become more frequent, the pathogen can maintain its reproductive success with less horizontal transmission, which translates to lower $\alpha^{*}$.

Adjusting the parameters of vertical transmission, v, and birth rate, $b_{s}$, impacts stable recovery rate, $\gamma^{*}$, as well. We subdivide the effects here into three groups.

First, when v is fixed at a low value, increasing $b_{s}$ lowers recovery rate, $\gamma^{*}$ (Figure 3). As mentioned above, larger reproductive rates are compensated for by a higher background mortality rate, which leads to a shorter host lifespan. Because of the trade‐off with birth rate while infected, the lower recovery rate allows the host to maintain its lifetime reproductive success in a shorter period. Notably, the reduction in $\gamma^{*}$ here is accompanied by an increase in $\alpha^{*}$ (Figure 3). The lower value of n explains this. Recall that when n is small, the risk associated with infection is low. Thus, the host is less concerned by the threat of the disease and more concerned with a reduction in fitness connected to the density of the population to which it belongs. Given that v is low, the host is similarly less concerned by the risks of producing low‐quality infected offspring.

Second, when v is fixed at a high value, an increase in $b_{s}$ boosts $\gamma^{*}$ (Figure 3). Now, an infected host prioritizes recovery because otherwise, it will transmit its infection to a significant fraction of its offspring. In other words, there is a shift toward enhancing the quality of reproductive output within a shortened life expectancy rather than the quantity.

Third, when v is increased, with $b_{s}$ held constant, we observe a reduction in recovery rate $\gamma^{*}$ (Figure 3). This is interpreted as a response to a decrease in pathogen‐induced mortality, $\alpha^{*}$, that is in turn driven by greater opportunities for vertical transmission.

### 3.3 Higher Profitability of Horizontal Transmission

**FIGURE 4:**
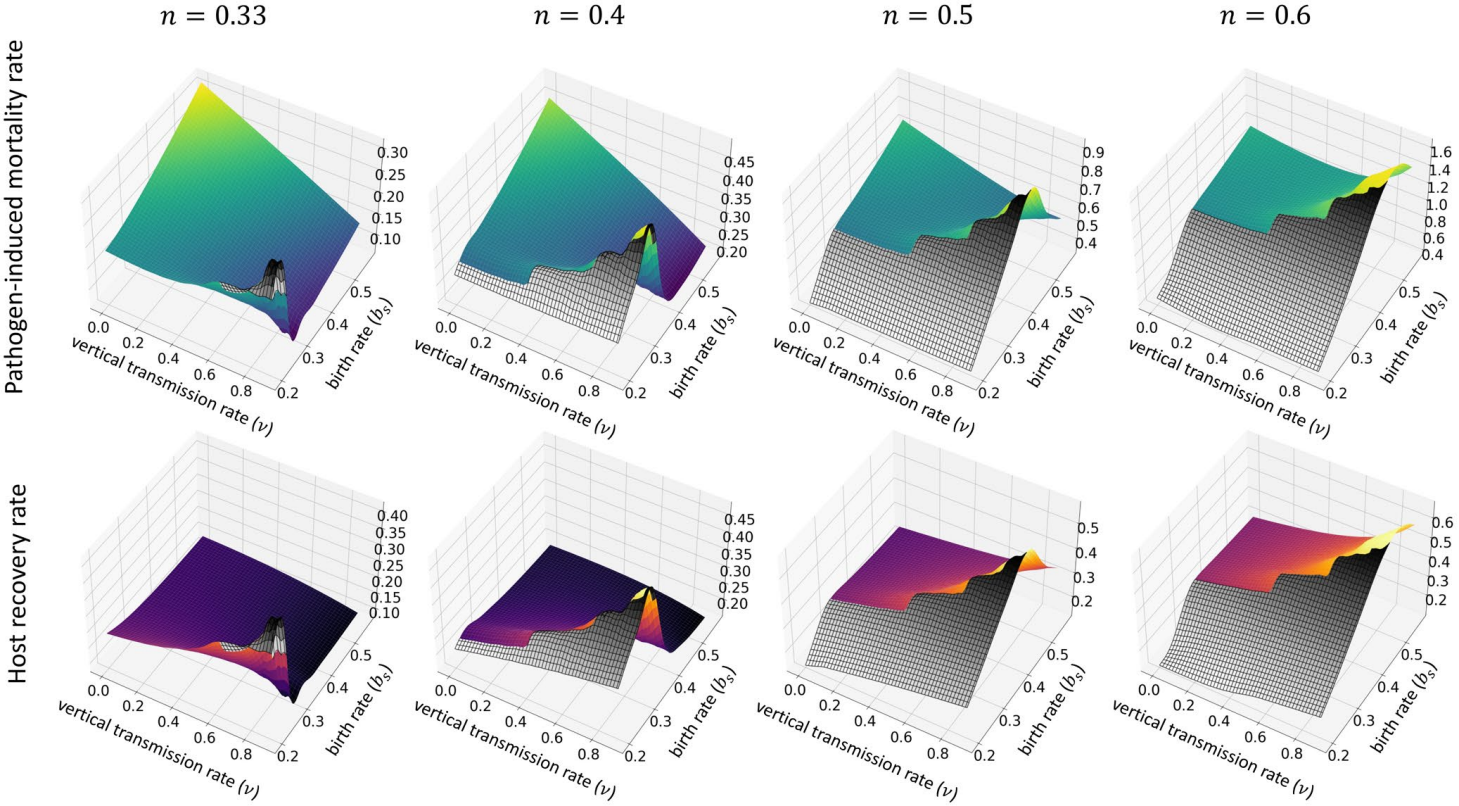
Variation in the evolutionarily stable values of the pathogen‐induced mortality rate $\left(\alpha^{*}\right)$ and the host recovery rate $\left(\gamma^{*}\right)$ in relation to different levels of vertical transmission rate $\left(v\right)$ and birth rate $\left(b_{s}\right)$ under distinct higher values of horizontal transmission profitability $\left(n\right)$, specifically for n=0.33,0.4,0.5, and 0.6. We observe a notable rise in these values at higher v levels. We assume the rate of exposed hosts becoming infectious, δ=1, and the background mortality rate, $\mu \left(N\right)=0.15N$, when N represents the total population. The gray wireframes represent the regions where the number of infections is effectively zero, so we consider the pathogen to be extinct. In addition, k=1 and c=1.

We now consider cases in which the profitability of horizontal transmission, n, is higher. In these cases, we see a deviation from the pattern established in the previous subsection (where n was lower). Specifically, the stable expressions show a noticeable local peak for higher rates of vertical transmission, v, and lower birth rate, $b_{s}$ (Figure 4). As an aside (for the moment), near the local peak, we lose the numerical data because our algorithm converges to an evolutionary equilibrium where there is effectively no infection. The loss of numerical data is evidenced by gray‐colored wire‐frames in Figure 4 where v is large and $b_{s}$ is relatively small. Biologically speaking, gray regions are where selection‐driven extinction of pathogens occurs.

The local peak we identify can be characterized in two distinct ways. First, for a fixed birth rate, $b_{s}$, increasing v eventually triggers a rise in the level of trait expression, that is, greater $\alpha^{*}$ and $\gamma^{*}$ (Figure 4). The intuition is that horizontal transmission is more profitable because we have a higher n, so the pathogen‐induced mortality is incentivized. It follows that the infected offspring produced by an infected host are at greater risk of pathogen‐induced mortality. From the host parent's perspective, the risk is amplified as rates of vertical transmission increase, resulting in a higher recovery rate. Of course, an increased recovery rate shortens the duration of infection, which in turn encourages even more horizontal transmission and greater pathogen‐induced mortality.

Second, for a fixed vertical transmission rate, v, increasing birth rate, $b_{s}$, precipitates a fall in the level of expression, at least near the peak (Figure 4). The intuition is that background mortality increases with birth rate, resulting in shorter host lifespan. Thus, increasing $b_{s}$ may prompt a reduction in $\gamma^{*}$ to preserve lifetime reproductive success over a shorter period. The changes we observe in the pathogen trait, $\alpha^{*}$, could be viewed as a co‐evolutionary response to the host. However, they do make sense on their own when we realize that increasing $b_{s}$ impacts the expected duration of infection through its relationship with the background mortality rate. As $b_{s}$ intensifies, infections become shorter‐lived, but opportunities for vertical transmission become more frequent as offspring are produced more quickly; this leads to the fall in $\alpha^{*}$ we see near the peak.

### 3.4 Virulence

We now explore the evolution of virulence defined in two distinct ways. The first definition of virulence we explore is related to case mortality, in other words, the probability of an infectious host's death due to its infection (Day 2002). The second definition is related to the fitness reduction experienced by a host when it acquires an infection (Read 1994).

**FIGURE 5:**
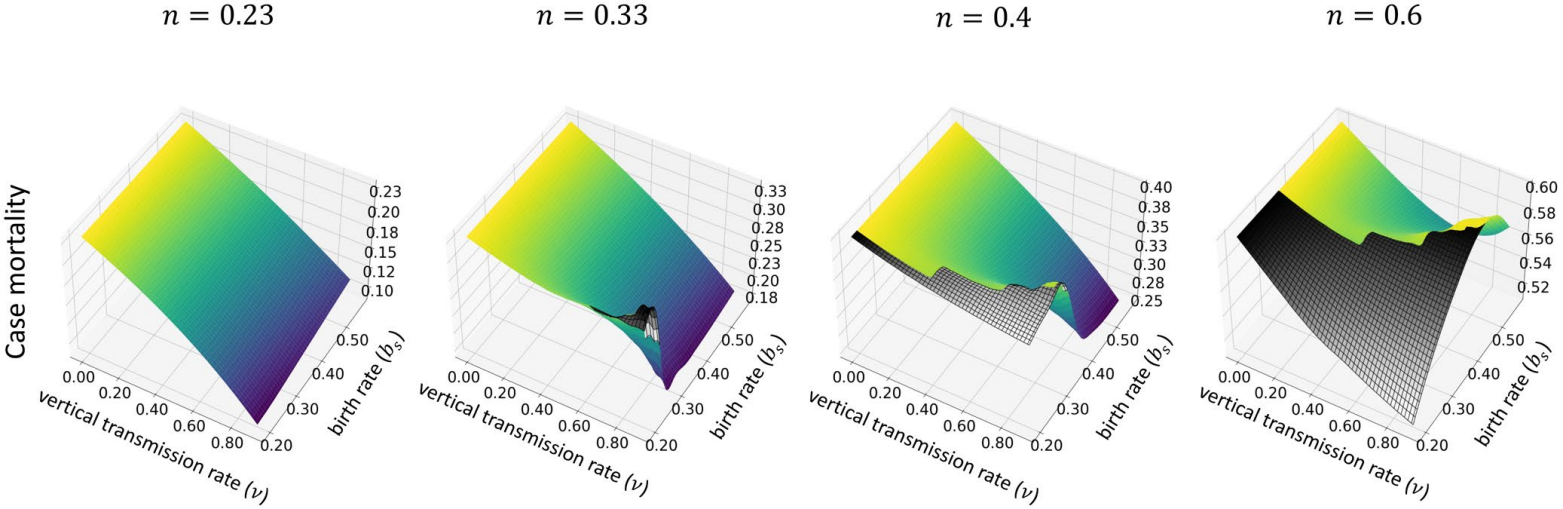
Variation in virulence as case mortality, which is calculated at co‐evolutionary equilibrium, across a range of vertical transmission $\left(v\right)$ and birth rates $\left(b_{s}\right)$, evaluated at different values of horizontal transmission profitability $\left(n\right)$, specifically for n=0.23,0.33,0.4, and 0.6. Each subplot represents how virulence adapts with increasing n. We assume the rate of exposed hosts becoming infectious, δ=1, and the background mortality rate, $\mu \left(N\right)=0.15\times N$. The gray wireframes represent the regions where the number of infections is effectively zero, so we consider the pathogen to be extinct.

Case mortality, the probability of an infectious host dying from its infection, is calculated as

(9) $$\frac{\alpha^{*}}{\alpha^{*}+\gamma^{*}+\mu \left(\bar{N}\right)},$$

where the superscript ‘
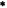
’ reminds us that the expression is evaluated at the co‐evolutionary equilibrium. Figure 5 shows how virulence, measured as case mortality, changes with the birth rate, $b_{s}$, vertical transmission rate, v, and horizontal transmission profitability, n. The relationship between case mortality and model parameters closely resembles that between pathogen‐induced mortality, $\alpha^{*}$, and model parameters (compare Figure 5 to Figures 3 and 4).

There are two additional noteworthy observations to be made about case mortality. First, it can increase as the vertical transmission rate, v, rises. Because the highest case mortality occurs when (but not only when) there is no vertical transmission, any such rise in case mortality is preceded by a reduction (Figure 5). This first noteworthy observation is consistent with the findings of Shillcock et al. (2023) in a model with no self‐regulation of the host population. It is inconsistent with the findings of Brotman and Wild (2025), whose model with density‐dependent host reproduction predicted a uniform negative relationship between virulence and vertical transmission rate.

The second noteworthy observation is that when there is no vertical transmission, the case mortality at evolutionary equilibrium equals the profitability of horizontal transmission, captured by the parameter n. In other words, the evolution of case mortality is completely determined by the shape of the trade‐off between the rates of horizontal transmission and pathogen‐induced mortality when v=0. The result, here, is due to the functional form we chose for $\beta \left(\alpha \right)$ (see also Day and Burns 2003). It is easy to prove, mathematically, that case mortality must equal n given our model for $\beta \left(\alpha \right)$ and given that the pathogen expresses ‘α’ at an evolutionary equilibrium level (Brotman and Wild 2025).

The second definition of virulence we consider is the reduction in fitness experienced by a host that moves from the susceptible category to the exposed category. In Appendix C, we show that virulence in the second sense is expressed as

(10) $$\frac{b_{s}-\mu \left(\bar{N}\right)}{\beta \left(\alpha^{*}\right)\bar{I}}.$$

This mathematical definition of virulence differs from the analogous one found in Shillcock et al. (2023) and in Brotman and Wild (2025). However, the difference is not related to the presence or absence of self‐regulation of the host population. It is related to the absence of an exposed class of host in previous work.

**FIGURE 6:**
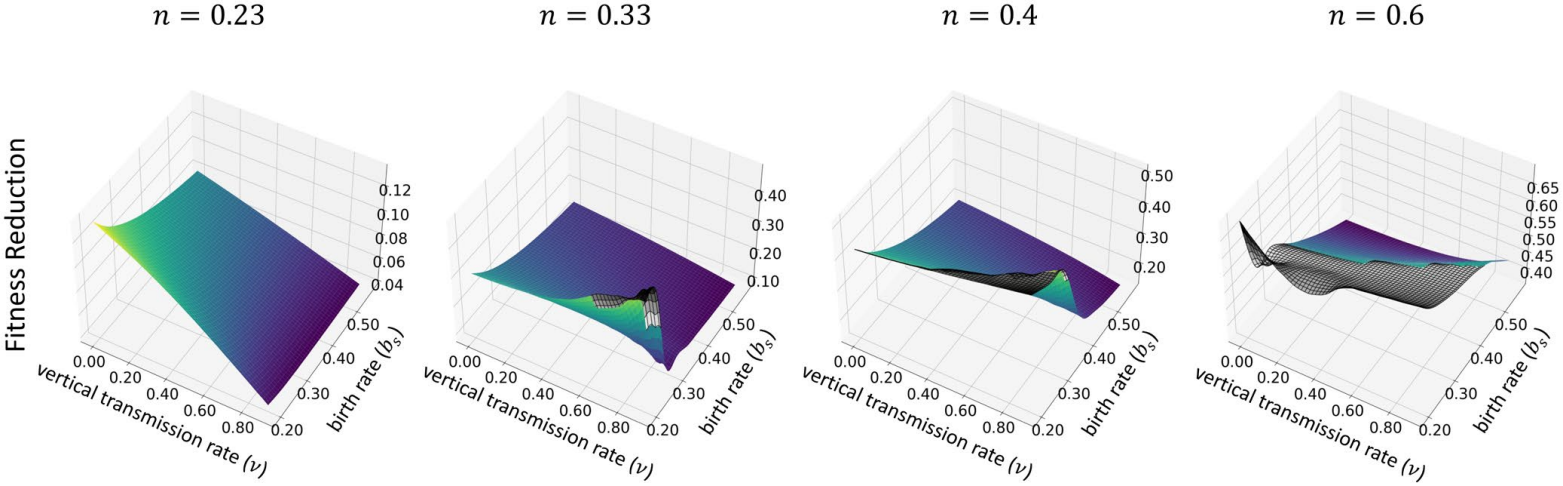
Variation in virulence as fitness reduction across a range of vertical transmission $\left(v\right)$ and birth rates $\left(b_{s}\right)$, evaluated at different values of horizontal transmission profitability $\left(n\right)$, specifically for n=0.23,0.33,0.4, and 0.6. Each subplot represents how virulence adapts with increasing n. We assume the rate of exposed hosts becoming infectious, δ=1, and the background mortality rate, $\mu \left(N\right)=0.15\times N$, when N represents the total population. The gray wireframes represent the regions where the number of infections is effectively zero, so we consider the pathogen to be extinct.

Despite the differences between our mathematical definition of virulence (in the sense of fitness reduction) and that of Shillcock et al. (2023), our qualitative predictions match theirs. In particular, virulence can increase with increasing vertical transmission, and this increase can lead to virulence exceeding the levels at which it is observed that vertical transmission is absent (Figure 6). We also note that our findings differ from those arising from the model studied by Brotman and Wild (2025) that implemented host self‐regulation by assuming density‐dependent birth.

## 4 Discussion

The co‐evolution of a pathogen and its host when the former is transmitted both vertically and horizontally has received recent attention (Mitchell et al. 2022; Shillcock et al. 2023; Brotman and Wild 2025). Previous work produced conflicting qualitative results on the effect that vertical transmission has on pathogen virulence. Shillcock et al. (2023) demonstrated that vertical transmission can select for more virulent pathogens, whereas Brotman and Wild (2025) showed that vertical transmission selects for reduced virulence. The differences, of course, stem from the assumptions these authors made. Shillcock et al. (2023) assumed that co‐evolving hosts pay the cost of immune function only when they are infected; they also assumed that the pathogen alone limits the growth of the host population. By contrast, Brotman and Wild (2025) assumed hosts pay the cost of immune function whether they are infected or not. Moreover, the growth of the host population in their model cannot persist above carrying capacity even when the pathogen is absent.

The conflicting results of previous authors (Shillcock et al. 2023; Brotman and Wild 2025) led us to ask, can vertical transmission promote pathogen virulence when a co‐evolving host pays the cost of immune function only when sick *and* experiences density‐dependent growth absent the pathogen? We find that the answer to the question is yes. Like previous authors (Shillcock et al. 2023), we find a positive relationship between the rate of vertical transmission and virulence is more likely when (a) host exploitation pays high dividends in terms of greater horizontal transmission (i.e., horizontal transmission is highly profitable), and (b) host birth rates are low.

The possibility of a positive relationship between pathogen virulence and vertical transmission seems surprising given the common theoretical expectation that vertical transmission incentivizes reduced host harm (Ewald 1994; Frank 1996; Lipsitch et al. 1996; Stearns and Medzhitov 2015; Cressler et al. 2016; Úbeda and Jansen 2016). However, these expectations are predicated on the assumption that the host's response to the pathogen does not co‐evolve. When the host can respond, things change. Because increased vertical transmission implies that an infected host produces more low‐quality (i.e., infected) offspring, raising the vertical transmission rate can incentivize an infected host to clear a pathogen more quickly (Shillcock et al. 2023). A more aggressive response mounted by the host can lead the pathogen to reciprocate, creating more infections horizontally (Shillcock et al. 2023). The escalating co‐evolutionary hostilities we describe here are not guaranteed, of course. As mentioned, we find that profitable host exploitation combined with low host birth rates sets the stage for escalation. This finding indicates that escalation is triggered by rising vertical transmission rates when the pathogen can make relatively large fitness gains through horizontal transmission (profitable exploitation implies substantial gains through horizontal infection, and low birth rate means fewer chances to pass to host offspring), and the host can little afford to produce infected offspring. The former point, at least, is reflected by empirical work with paramecia showing that their benign vertically transmitted bacterial parasites were more successful when the paramecium reproduction rate was high (Kaltz and Koella 2003).

Even when the qualitative relationship between vertical transmission rate and virulence is negative, we find that highly profitable horizontal transmission raises the level of pathogen‐induced mortality. This agrees with patterns revealed in the work of Brotman and Wild (2025) and others (e.g., Day and Burns 2003). Indeed, a general prediction of our model is that pathogen‐induced mortality and overall virulence increase when horizontal transmission becomes more profitable for the pathogen. This prediction is analogous to ones made about the impact of so‐called ‘imperfect vaccines’ on pathogen evolution. An imperfect vaccine reduces the overall costs of virulence to pathogens, for example, by lowering the mortality rate without limiting horizontal transmission (Gandon et al. 2001; Read et al. 2015; Fleming‐Davies et al. 2018). In other words, an imperfect vaccine provides a pathogen with an incentive to exploit its host to a greater degree. In the context of our model, such an incentive arises as we increase the parameter n. Increasing this parameter raises the benefits of host exploitation, holding the cost fixed.

Our prediction that virulence rises as horizontal transmission becomes more profitable is also consistent with experimental findings. Specifically, it is consistent with experimental reductions in virulence that emerge when horizontal transmission becomes less profitable because it is prevented (Bull et al. 1991). It is also consistent with experimental results of Stewart et al. (2005) and Pagán et al. (2014) who found a positive relationship between horizontal transmission and pathogen virulence. Pagán et al. (2014) also demonstrated that plant viruses transmitted horizontally evolved to be more virulent than those transmitted vertically.

We identified co‐evolutionary outcomes where both host recovery and pathogen‐induced mortality rate are low. In those cases, the infection occurs at numbers that are positive but effectively zero. We interpret this result as selection‐driven extinction of the pathogen because the low number of infectious hosts implies an elevated risk of stochastic loss of the pathogen (Dieckmann and Metz 2006). Our interpretation is also consistent with theory that argues that strict vertical transmission elevates extinction risk (see Cressler et al. 2016), since low pathogen‐induced mortality in our model implies horizontal transmission is low (nearly strict vertical transmission). In keeping with other theory on host‐pathogen co‐evolution (van Baalen 1998), we find that density‐dependent regulation of the host population growth enables the host to drive the pathogen to extinction without succumbing to extinction itself. One might argue that density dependence is key to achieving selection‐driven extinction because it implies that the invasion fitness of both the host and the pathogen depends on resident traits. As previously argued, when invasion fitness is independent of resident traits—as it is in the model developed by Shillcock et al. (2023)—selection maximizes the population mean fitness, making selection‐driven extinction impossible (Matsuda and Abrams 1994; but see Parvinen and Dieckmann 2013, for clarity on selection‐driven extinction under optimizing selection). Nevertheless, we cannot discount the role played by our decision to limit the cost of immune function to infected hosts only. When susceptible hosts do not bear the cost of immune function, they act as a demographic reservoir, rescuing birth rates when their infected counterparts incur the heavy cost of eliminating the pathogen. This rescue effect likely enhances the host population's ability to persist.

Unlike previous work (van Baalen 1998), our model allows for vertical transmission of the pathogen. Clearly, then, vertical transmission is not necessary for selection‐driven extinction of the pathogen to occur. However, our results show that selection‐driven extinction of the pathogen does occur more readily when vertical transmission leads to high virulence, measured as a reduction in host fitness in a resident population. In other words, vertical transmission is more strongly associated with selection‐driven extinction of the pathogen when the fitness reduction experienced by infected hosts is high. This, in turn, is indicative of a greater competitive ecological advantage for susceptible hosts. Previous ecological models have predicted that susceptible hosts can out‐compete infected ones, leading to the extinction of vertically transmitted parasites (Hochberg 1991). Our work shows that similar predictions arise as a result of co‐evolutionary forces.

Empirical evidence has pointed to the existence of trade‐offs between horizontal and vertical transmission (Turner et al. 1998; Kover and Clay 1998; Kaltz and Koella 2003). Indeed, it is often assumed that horizontal and vertical transmission either trade off directly or have opposing indirect effects on host fitness (Antonovics et al. 2017). While our model did not force a trade‐off between horizontal and vertical transmission, their indirect effects sometimes created a trade‐off or the appearance thereof. First, a pathogen with a greater rate of horizontal transmission reduces the expected lifespan of its host, which in turn reduces its ability to create new infections vertically. Second, we found that there is sometimes an elevated risk of pathogen extinction when rates of vertical transmission are high and horizontal transmission is profitable. This second finding suggests that the co‐occurrence of high rates of horizontal transmission and high rates of vertical transmission should be rare in nature. That said, we are reluctant to frame this second prediction as a trade‐off per se, as it is a population‐level feature rather than a constraint imposed on individuals.

There is no doubt that trade‐offs feature prominently in this work. While we have offered justification for the trade‐offs built into our co‐evolutionary model, different host trade‐offs can yield different results (Cousineau and Alizon 2014). We assumed that hosts trade reproductive success for recovery; however, it is a sad fact that recovery is sometimes not possible. Future work may wish to explore different host trade‐offs to deal with persistent infections. One possibility is that hosts increase reproduction to incentivize (presumably less harmful) vertical transmission but suffer greater mortality as a result. Exactly how the pathogen would respond to enhanced opportunities for vertical transmission during shorter periods of infection is not clear, but it would be interesting to explore.

Our findings are likely also a function of the modeling framework we adopted. We used a framework that emphasizes equilibrium dynamics and genetically homogeneous populations: we expressed mutant fitness in the background of a steady‐state population and looked for monomorphic co‐evolutionary outcomes that resist further change. The same framework was adopted by the previous work upon which we have built (Úbeda and Jansen 2016; Shillcock et al. 2023; Brotman and Wild 2025). We could have modeled host‐pathogen co‐evolution using a “gene‐for‐gene” framework where genetic polymorphism in the host and pathogen populations is potentially more substantial (Jayakar 1970). In this alternative framework, specific host genotypes resist specific pathogen genotypes, allowing co‐evolutionary cycles to emerge (e.g., Sasaki 2000). How these non‐equilibrium dynamics might be impacted by vertical transmission and how we might quantify overall virulence is unclear, given that our focus was not on gene‐for‐gene scenarios. Future work that assesses the impact of vertical transmission on gene‐for‐gene systems would be valuable because (a) noteworthy plant‐pathogen interactions can be characterized as gene‐for‐gene (Thompson and Burdon 1992) and (b) vertical transmission is an important route for some plant pathogens (Pagán 2022).

Our findings are certainly limited by our modeling assumptions. One key limitation of our model is the assumption that hosts can only be infected by a single strain of pathogen at any given time. Our model, therefore, does not account for the possibility of co‐infections with multiple pathogen strains. If co‐infecting strains did occur and were distantly related, we would expect even higher levels of pathogen trait expression to evolve because each strain would be more inclined to pursue selfish interests (Frank 1992). In this case, we also speculate that the level of host trait expression (recovery) would increase in response to a more aggressive disease‐causing agent.

Another key limitation of our model is the assumption that host mutations are independent of pathogen mutations. This means we neglect the potential for phenotypic correlations between individual lineages of hosts and individual lineages of pathogens to emerge. Positive phenotypic correlations between social partners—for example, a pathogen and its host– are known to produce cooperative evolutionary outcomes (Fletcher and Doebeli 2009). Thus, if we were to allow these correlations in our model, they would likely drive trait expressions to lower levels. Similar predictions are made by models built around sequential move evolutionary games that allow positive phenotypic correlations between host and pathogen to emerge (Taylor et al. 2006). Whether low levels of trait expressions in these cases lead to pathogen extinction is an open question.

Finally, we assumed that host population growth is regulated by density‐dependent background mortality. As we have explained, this modeling decision means that the duration of infection is sensitive to host demography. In other words, a particular life history feature of the pathogen is sensitive to changes in the host population size. Had we incorporated density‐dependent host birth rates instead, the duration of infection would have been independent of host population size. In this case, the incentives that shape the evolution of pathogen trait expression may be quite different. In addition, we have neglected the possibility of age‐related changes in host demographic rates, and accounting for these changes could further complicate predictions. Future work should focus on outlining the role of density‐dependent and age‐related demographic rates in shaping the co‐evolution of hosts and their vertically transmitted pathogens.

## Author Contributions

**Bita Ghodsi:** conceptualization (equal), formal analysis (equal), methodology (equal), software (lead), visualization (lead), writing – original draft (equal), writing – review and editing (equal). **Geoff Wild:** conceptualization (equal), formal analysis (equal), funding acquisition (lead), methodology (equal), supervision (lead), writing – original draft (equal), writing – review and editing (equal).

## Conflicts of Interest

The authors declare no conflicts of interest.

## Supporting information


**Data S1:** Supporting Information.

## Data Availability

The computer code used to generate numerical results has been included as a supplement to this manuscript (see Supporting Information).

## Appendix A Pathogen Fitness

We suppose a mutant pathogen strain that induces a mortality rate of $\alpha_{m}$ has arisen. We use $E_{m}=E_{m}\left(t\right)$ to denote the number of hosts infected with the mutant pathogen but not infectious, and we use $I_{m}=I_{m}\left(t\right)$ to denote the number of hosts infected with the mutant strain who are also infectious. If we assume co‐infections cannot occur, then the full ecological dynamics are given by

(A.1)

where N now denotes $S+E+I+E_{m}+I_{m}$, the total size of the host population.

We use the vector $\left(\bar{S},\bar{E},\bar{I}\right)$ to denote the endemic equilibrium that can be established in the absence of the mutant pathogen. That same equilibrium exists for Equation (A.1), but is expressed instead as $\left(\bar{S},\bar{E},\bar{I},0,0\right)$. By linearizing Equation (A.1) about this mutant‐free equilibrium, we obtain the approximate dynamics of the mutant pathogen while it is rare:

(A.2)
$$\left[\begin{array}{c} \frac{{\mathrm{dE}}_{m}}{\mathrm{dt}}\\ \frac{{\mathrm{dI}}_{m}}{\mathrm{dt}} \end{array}\right]=\left[\begin{array}{cc} -\left(\delta +\mu \left(\bar{N}\right)\right) & \beta \left(\alpha_{m}\right)\bar{S}+{\mathrm{vb}}_{I}\left(\gamma \right)\\ \delta & -\left(\gamma +\mu \left(\bar{N}\right)+\alpha_{m}\right) \end{array}\right]\left[\begin{array}{c} E_{m}\\ I_{m} \end{array}\right],$$

where $\bar{N}=\bar{S}+\bar{E}+\bar{I}$ represents the total population at the mutant‐free equilibrium.

We now apply the “next‐generation” approach to develop a mathematical expression of the pathogen invasion fitness (Hurford et al. 2010). We decompose the matrix in Equation (A.2) as $F_{p}-V_{p}$, where

(A.3)
$$F_{p}=\left[\begin{array}{cc} 0 & \beta \left(\alpha_{m}\right)\bar{S}+{\mathrm{vb}}_{I}\left(\gamma \right)\\ 0 & 0 \end{array}\right]\;\text{and}\;V_{p}=\left[\begin{array}{cc} \delta +\mu \left(\bar{N}\right) & 0\\ -\delta & \gamma +\alpha_{m}+\mu \left(\bar{N}\right) \end{array}\right].$$

The matrix $V_{p}$ is invertible and

(A.4)
$$V_{p}^{-1}=\frac{1}{\left(\delta +\mu \left(\bar{N}\right)\right)\left(\gamma +\mu \left(\bar{N}\right)+\alpha_{m}\right)}\left[\begin{array}{cc} \gamma +\mu \left(\bar{N}\right)+\alpha_{m} & 0\\ \delta & \delta +\mu \left(\bar{N}\right) \end{array}\right]$$

is its inverse.

We index the rows and columns of $F_{p}$ and $V_{p}^{-1}$ as i,j,ℓ=1,2, where category 1 refers to hosts exposed to the mutant pathogen strain and category 2 refers to infectious hosts carrying the mutant strain. Thus, the ijth entry of $F_{p}$ gives the rate at which a category j infection produces a new category i infection. The jℓth entry of $V_{p}^{-1}$ is the average length of time a current category ℓ infection spends as a category j infection during its lifetime. With this decomposition, then, the iℓth element of the matrix, $K_{p}=F_{p}V_{p}^{-1}$, is interpreted as the expected lifetime number of new type i infections produced by a given infection that is currently of type k. We call $K_{p}$ the “next generation matrix” for this reason.

The largest eigenvalue of the next‐generation matrix gives us the invasion fitness of the mutant pathogen in a resident pathogen population, denoted $W_{p}\left(\alpha_{m};\alpha ,\gamma \right)$. Given that $K_{p}$ is a triangular matrix, its largest eigenvalue is easily determined to be

(A.5)
$$W_{p}\left(\alpha_{m};\alpha ,\gamma \right)=\frac{\delta }{\delta +\mu \left(\bar{N}\right)}\frac{\beta \left(\alpha_{m}\right)\bar{S}+{\mathrm{vb}}_{I}\left(\gamma \right)}{\gamma +\mu \left(\bar{N}\right)+\alpha_{m}},$$

where $\bar{S}$ and $\bar{N}$ are functions of α and γ.

## Appendix B Host Fitness

We suppose a rare lineage of mutant hosts has arisen within a resident population at its endemic equilibrium. Infectious mutant hosts recover from infections at a rate of $\gamma_{m}$. We use $S^{m}=S^{m}\left(t\right)$, $E^{m}=E^{m}\left(t\right)$ and $I^{m}=I^{m}\left(t\right)$ to denote the number of susceptible, exposed, and infectious mutant hosts, respectively. We assume that only the resident pathogen strain is circulating in the resident population. We also treat the host as haploid and asexual, so the full dynamics of this new ecological system are given by

(B.1)

where $N=S+E+I+S^{m}+E^{m}+I^{m}$ now represents the total population of resident and mutant hosts.

Equation (B.1) admits an equilibrium free of the mutant host, $\left(\bar{S},\bar{E},\bar{I},0,0,0\right)$. We linearize about this mutant‐free equilibrium to obtain the approximate dynamics of the mutant host while it is rare:

(B.2)
$$\left[\begin{array}{c} \frac{{\mathrm{dS}}^{m}}{\mathrm{dt}}\\ \frac{{\mathrm{dE}}^{m}}{\mathrm{dt}}\\ \frac{{\mathrm{dI}}^{m}}{\mathrm{dt}} \end{array}\right]=\left[\begin{array}{ccc} b_{s}-\beta \left(\alpha \right)\bar{I}-\mu \left(\bar{N}\right) & b_{s} & \left(1-v\right)b_{I}\left(\gamma_{m}\right)+\gamma_{m}\\ \beta \left(\alpha \right)\bar{I} & -\delta -\mu \left(\bar{N}\right) & {\mathrm{vb}}_{I}\left(\gamma_{m}\right)\\ 0 & \delta & -\left(\gamma_{m}+\alpha +\mu \left(\bar{N}\right)\right) \end{array}\right]\left[\begin{array}{c} S^{m}\\ E^{m}\\ I^{m} \end{array}\right].$$

We decompose the matrix in the previous line as $F_{h}-V_{h}$, where

(B.3)
$$F_{h}=\left[\begin{array}{ccc} b_{s} & b_{s} & \left(1-v\right)b_{I}\left(\gamma_{m}\right)\\ 0 & 0 & {\mathrm{vb}}_{I}\left(\gamma_{m}\right)\\ 0 & 0 & 0 \end{array}\right],V_{h}=\left[\begin{array}{ccc} \beta \left(\alpha \right)\bar{I}+\mu \left(\bar{N}\right) & 0 & -\gamma_{m}\\ -\beta \left(\alpha \right)\bar{I} & \delta +\mu \left(\bar{N}\right) & 0\\ 0 & -\delta & \gamma_{m}+\alpha +\mu \left(\bar{N}\right) \end{array}\right].$$

The matrix $F_{h}$ captures mutant host reproductive rates, and the inverse of $V_{h}$ (not shown) captures the expected amount of time a host remains in a given state (Hurford et al. 2010). We denote the next generation matrix for the host as $K_{h}=F_{h}V_{h}^{-1}$, and its spectral radius gives us the host invasion fitness, $W_{h}\left(\gamma_{m};\alpha ,\gamma \right)$. This spectral radius appears in Equation (4) of the main text.

## Appendix C Virulence as Host Fitness Reduction

Here, we derive an expression for virulence as a measure of the fitness reduction experienced by a host that moves from the susceptible category to the exposed category at equilibrium. Let $W_{S}$, $W_{E}$, and $W_{I}$ denote the fitness of susceptible, exposed, and infectious hosts, respectively. The vector $\left(W_{S},W_{E},W_{I}\right)$ will be the left eigenvalue of the matrix

(C.1)
$$\left[\begin{array}{ccc} b_{s}-\beta \left(\alpha \right)\bar{I}-\mu \left(\bar{N}\right) & b_{s} & \left(1-v\right)b_{I}\left(\gamma \right)+\gamma \\ \beta \left(\alpha \right)\bar{I} & -\delta -\mu \left(\bar{N}\right) & {\mathrm{vb}}_{I}\left(\gamma \right)\\ 0 & \delta & -\left(\gamma +\alpha +\mu \left(\bar{N}\right)\right) \end{array}\right]$$

associated with the zero eigenvalue (Shillcock et al. 2023). Note that the matrix in (C.1) is identical to the one that appears in Equation (B.2), except $\gamma_{m}$ in (B.2) is replaced by γ in (C.1). The fact that the matrix in (C.1) has a zero eigenvalue is connected to the fact that $\gamma_{m}$ has been set equal to γ: selection cannot distinguish between hosts.

We set $W_{S}=1$ without loss of generality. We then apply $\left(1,W_{E},W_{I}\right)$ to the left of the first column in the matrix in (C.1) and use the fact that the vector in question is an eigenvector (associated with an eigenvalue of zero) to obtain

(C.2)
$$b_{s}-\mu \left(\bar{N}\right)-\beta \left(\alpha \right)\bar{I}+W_{E}\;\beta \left(\alpha \right)\bar{I}=0.$$

Solving for $W_{E}$ we conclude that $W_{E}=\frac{\beta \left(\alpha \right)\bar{I}-\left(b_{s}-\mu \left(\bar{N}\right)\right)}{\beta \left(\alpha \right)\bar{I}}$ which is positive and less than 1. The fitness reduction experienced by a susceptible host who becomes exposed is

(C.3)
$$1-W_{E}=\frac{b_{s}-\mu \left(\bar{N}\right)}{\beta \left(\alpha \right)\bar{I}},$$

which agrees with the expression that appears in the main text.
