# Supplementary material for: Virulence and Vertically Transmitted Pathogens: The Role of Costs Paid by Co‐Evolving Hosts in a Self‐Regulating Population
Source: Ecol Evol. 2025 Jul 31;15(8):e71840. doi: 10.1002/ece3.71840 (PMC12313842; doi:10.1002/ece3.71840)
Supplement: Supplementary file 1 — Data S1: Supporting Information. [file ECE3-15-e71840-s001.pdf]

Supplement to, "VIRULENCE AND VERTICALLY TRANSMITTED PATHOGENS: THE ROLE OF COSTS PAID BY CO-EVOLVING HOSTS IN A SELF-REGULATING POPULATION"

Bita Ghodsi and Geoff Wild

Department of Mathematics, Western University, London, Ontario, Canada

1. Jupyter notebook implementation

This supplement contains the Jupyter Notebook, which includes the complete implementation details of the methods and analyses discussed in the second chapter. Each step of the implementation is thoroughly documented with code snippets and explanations to enhance understanding and reproducibility.

6

0.0.1 Libraries

In the following cell, we import essential libraries for numerical operations, data handling, and visualization.

[2]:
```
import numpy as np
from scipy.integrate import odeint
import matplotlib.pyplot as plt
import pandas as pd
import csv
from scipy.interpolate import griddata
from mpl_toolkits.mplot3d import Axes3D
```

0.0.2 Notation Key

alpha: pathogen_induced mortality rate (resident)
gamma: host recovery rate (resident)
alpha_m: pathogen-induced mortality rate (mutant)
gamma_m: host recovery rate (mutant)
bs: birth rate of non-infectious individuals
mu: background mortality rate
mu_coef(μ_0): coefficient of background mortality rate
beta: horizontal transmission rate constant
delta: the rate at which the exposed population gets infectious
N: total population
bI: birth rate of infectious individuals
n: profitability of the horizontal transmission to the pathogen
S, E, I: susceptible, exposed, and infectious population size
z : (S, E, I)
v: vertical transmission rate
Wp: pathogen fitness function
Wh: host fitness function
eps: epsilon
niter: number of iteration

0.0.3 Defining Key Functions

In the following code snippet, we define several crucial functions required for simulating and analyzing the dynamics of our model.

- **`bI(gamma, bs)`**: Calculates the birth rate of the infectious population based on the host recovery rate (γ) denoted by `gamma` and the basic birth rate (b_s) denoted by `bs`.
- **`mu(N, mu_coef = 0.15)`**: Computes the background mortality rate as a function of the total population (`N`) scaled by a coefficient, μ_0, represented by `mu_coef`.
- **`beta(alpha, n)`**: Determines the horizontal transmission rate constant, as a function of the pathogen-induced mortality rate ('α'), denoted by `alpha`, and `n` (which represents the profitability of the horizontal transmission to the pathogen).
- **`population_rates(z, gamma, alpha, bs, v, n, delta=1, mu_coef = 0.15)`**: Represents the population model, calculating the rate of change of susceptible (`S`), exposed (`E`), and infectious (`I`) population sized over time given a set of parameters.
- **`endemic_equilibrium(z, gamma, alpha, bs, v, n)`**: Returns the endemic equilibrium of the population, by an iterative process of updating $\frac{dz}{dt}$ and adjusting `z` based on that so that the magnitude of the gradient falls below a specified threshold, indicating convergence to an optimal point. The rates of change are calculated at each iteration using the `population_rates` function.
- **`Wh(z, gamma_m, alpha, bs, v, n, delta=1)`** and **`Wp(z, gamma, alpha_m, bs, v, n, delta=1)`**: Calculate the fitness of the host and the pathogen, respectively, given sets of parameters.
- **`gradient_fitness_pathogen(z, gamma, alpha, bs, v, n, eps)`** and **`gradient_fitness_host(z, gamma, alpha, bs, v, n, eps)`**: Return the approximate gradients of the fitness functions, evaluated at the input parameters `gamma` and `alpha`, using the other provided parameter values.
- **`convergence_stable(z, gamma, alpha, bs, v, n)`**: Starting with an initial guess for stable α and γ, the endemic equilibrium is found using the `endemic_equilibrium` function. Then, it evaluates the fitness of the pathogen and the host at equilibrium using the `Wp` and `Wh` functions, respectively. Then, it calculates the fitness gradients. This iterative process continues, with updates to the evolutionary trait expressions, until the gradients approach a tolerance level near zero. The procedure returns the convergence stable γ and α, along with other information, based on a given set of parameters.
- **`ESS(gradient_fitness_pathogen, gradient_fitness_host)`**: Evaluate whether both fitness gradients are less than zero to confirm the presence of an Evolutionarily Stable Strategy (ESS). It returns 1 if an ESS is confirmed and 0 otherwise.

[8]:
```
# Function to calculate the adjusted birth rate of infectious individuals
def bI(gamma, bs):
    return bs - (gamma**2)

# Function to calculate the background mortality rate based on total population
def mu(N, mu_coef=0.15):
    return mu_coef * N

# Function to calculate the rate constant of horizontal transmission
def beta(alpha, n):
```

```python
    return alpha ** n

# Main function to calculate the rates of change in population classes
def population_rates(z, gamma, alpha, bs, v, n, delta=1, mu_coef=0.15):
    N = np.sum(z)  # Summing up the population to get total N
    mu_val = mu(N)
    beta_val = beta(alpha, n)
    bI_val = bI(gamma, bs)

    # Differential equations representing the changes in susceptible, exposed, and infectious populations
    dSdt = bs * z[0] + bs * z[1] + (1 - v) * bI_val * z[2] - beta_val * z[2] * z[0] + gamma * z[2] - mu_val * z[0]
    dEdt = beta_val * z[2] * z[0] + v * bI_val * z[2] - delta * z[1] - mu_val * z[1]
    dIdt = delta * z[1] - gamma * z[2] - alpha * z[2] - mu_val * z[2]

    dzdt = np.array([dSdt, dEdt, dIdt])
    return dzdt

# Function to find the equilibrium state of the population
def endemic_equilibrium(z, gamma, alpha, bs, v, n):
    N = np.sum(z)
    dzdt = np.array([1.0, 0.01, 0.0]) #initializing dz/dt

    # Iteratively finding the equilibrium state until changes are below the threshold
    while np.max(np.abs(dzdt)) > 1e-05:
        dzdt = population_rates(z, gamma, alpha, bs, v, n)
        z += 0.01 * dzdt

    return z

# Function to calculate host fitness with mutant recovery rate
def Wh(z, gamma_m, alpha, bs, v, n, delta=1):
    N = np.sum(z)
    beta_val = beta(alpha, n)
    bI_val = bI(gamma_m, bs)
    mu_val = mu(N)

    F = np.array([[bs, bs, (1-v) * bI_val],
                  [0, 0, v * bI_val],
                  [0, 0, 0]])
    V = np.array([[z[2] * beta_val + mu_val, 0, -gamma_m],
                  [-z[2] * beta_val, delta + mu_val, 0],
                  [0, -delta, alpha + gamma_m + mu_val]])
```

```python
    K = np.matmul(F, np.linalg.inv(V))
    return ((K[0][0] + K[1][1]) + np.sqrt((K[0][0] + K[1][1])**2 - 4 * (K[0][0] * K[1][1] - K[1][0] * K[0][1]))) / 2

# Function to calculate pathogen fitness with mutant mortality rate
def Wp(z, gamma, alpha_m, bs, v, n, delta=1):
    N = np.sum(z)
    beta_val = beta(alpha_m, n)
    bI_val = bI(gamma, bs)
    mu_val = mu(N)

    return ((delta) / (delta + mu_val)) * (((beta_val * (z[0]) + v * bI_val)) / (gamma + alpha_m + mu_val))

# Function to test convergence and stability of the system
def convergence_stable(z, gamma, alpha, bs, v, n):
    niter = 0
    tol = 1e-06
    eps = 0.0001

    while True:
        N = np.sum(z)
        niter += 1
        mu_val = mu(N)
        z = endemic_equilibrium(z, gamma, alpha, bs, v, n)

        Wh_up = Wh(z, gamma + eps, alpha, bs, v, n)  #Wh at gamma_m = gamma + eps
        Wh_down = Wh(z, gamma - eps, alpha, bs, v, n) #Wh at gamma_m = gamma - eps
        Gh = (Wh_up - Wh_down) / (2 * eps)

        Wp_up = Wp(z, gamma, alpha + eps, bs, v, n) #Wp at alpha_m = alpha + eps
        Wp_down = Wp(z, gamma, alpha - eps, bs, v, n) #Wp at alpha_m = alpha - eps
        Gp = (Wp_up - Wp_down) / (2 * eps)

        #updating alpha & gamma using Gp & Gh
        gamma += 0.01 * Gh
        alpha += 0.01 * Gp

        if abs(Gh) < tol and abs(Gp) < tol:
            break

    return gamma, alpha, Gp, Gh, z
```

```
# Function to calculate the gradient of fitness for the host using the finite␣
↪different approximation
def gradient_fitness_host(z, gamma, alpha, bs, v, n, eps):
    return (Wh(z, gamma + 2 * eps, alpha, bs, v, n) - 2 * Wh(z, gamma, alpha,␣
↪bs, v, n) +
            Wh(z, gamma - 2 * eps, alpha, bs, v, n)) / (4 * (eps) ** 2)

# Function to calculate the gradient of fitness for pathogen using the finite␣
↪different approximation
def gradient_fitness_pathogen(z, gamma, alpha, bs, v, n, eps):
    return (Wp(z, gamma, alpha + 2 * eps, bs, v, n) - 2 * Wp(z, gamma, alpha,␣
↪bs, v, n) +
            Wp(z, gamma, alpha - 2 * eps, bs, v, n)) / (4 * (eps) ** 2)

# Function to determine if an evolutionarily stable strategy (ESS) is achieved
def ESS(gradient_fitness_pathogen, gradient_fitness_host):
    if gradient_fitness_pathogen < 0 and gradient_fitness_host < 0:
        return 1
    else:
        return 0
```

0.0.4 Creating CSV Data file

In the following code snippet, we first create a CSV file named `model_data.csv` with defined headers. We then loop over values of `bs`, `v`, and `n` values, adding records to the CSV file. (As the code execution is time-consuming and may be interrupted, I executed the code separately for each `n` value.)

```
[ ]: # defining headers
headers = ["bs", "delta", "v", "gamma*", "alpha*", "S_bar", "E_bar","I_bar",␣
↪"N", "n", "beta", "bI", "Gp", "Gh", "mu", "ESS"]
# Create the CSV file and write headers
with open('model_data.csv', 'w', newline='') as file:
    writer = csv.writer(file)
    writer.writerow(headers)


# Loop over the specified n values
for n in [0.23, 0.27, 0.33, 0.36, 0.4, 0.5, 0.6, 0.7]:
    for bs in np.arange(0.2, 0.6, 0.04):
        for v in np.arange(0.0, 0.1, 0.05):

            gamma = 0.1
            alpha = 0.1
            eps_val = 0.001
            z = np.array([0.6, 0.2, 0.7])
```

```
            # Compute convergence stable and gradient fitness
            gamma, alpha, Gp, Gh, z = convergence_stable(z, gamma, alpha, bs,␣
↪v, n)
            gradient_fitness_pathogen_val = gradient_fitness_pathogen(z, gamma,␣
↪alpha, bs, v, n, eps_val)
            gradient_fitness_host_val = gradient_fitness_host(z, gamma, alpha,␣
↪bs, v, n, eps_val)

            # Determine if an Evolutionarily Stable Strategy (ESS) is achieved
            ESS_val = ESS(gradient_fitness_pathogen_val,␣
↪gradient_fitness_host_val)

            # Compute the total population at ESS
            N = z[0] + z[1] + z[2]

            # open the dataset and add a row
            with open('model_data.csv', 'a', newline='') as file:
              writer = csv.writer(file)
              writer.writerow([round(bs, 3), 1, round(v, 3), gamma, alpha,␣
↪z[0], z[1], z[2], N, round(n, 3), beta(alpha, n), bI(gamma, bs), Gp, Gh ,␣
↪mu(N), ESS_val])
```

0.0.5 Importing Data/ Adding Virulence

In the following code snippet, we import the dataset and augment it by adding new columns for case mortality and virulence (fitness reduction).

[5]:
```
import pandas as pd

# Load the dataset from a CSV file
df = pd.read_csv('model_data.csv')

# Compute fitness reduction as a new column named 'virulence'
df['virulence'] = (df["bs"] - df["mu"])/(df["beta"] * df["I_bar"])

# Compute case mortality rate and add it as a new column named 'case_mortality'
df['case_mortality'] = df['alpha*'] / (df['alpha*'] + df['gamma*'] + df['mu'])
```

0.0.6 Visualization

Here, we visualize the effects of variation in `n` (horizontal transmission profitability) on the pathogen-induced mortality rate and host recovery rate across different values of `v` (vertical transmission rate) and `bs` (birth rate).

[13]:
```
# Pre-defined values of 'n' for which analysis is conducted
n_values = [0.33, 0.4, 0.5, 0.6, 0.7]
```

```
# Filtering the dataframe for each 'n' value to create separate dataframes
df_beta023 = df[df['n'] == 0.23]
df_beta027 = df[df['n'] == 0.27]
df_beta030 = df[df['n'] == 0.3]
df_beta033 = df[df['n'] == 0.33]
df_beta036 = df[df['n'] == 0.36]
df_beta040 = df[df['n'] == 0.4]
df_beta050 = df[df['n'] == 0.5]
df_beta060 = df[df['n'] == 0.6]
df_beta070 = df[df['n'] == 0.7]

# Selecting dataframes for the analysis
dfs = [df_beta033, df_beta040, df_beta050, df_beta060, df_beta070]

# Setting up the figure for 3D plotting
fig = plt.figure(figsize=(70, 20))

# Loop through each selected dataframe and corresponding 'n' value
for i, (n, df_filtered) in enumerate(zip(n_values, dfs), start=1):
    # Create grid points for interpolation based on 'v' and 'bs' ranges
    xi = np.linspace(df_filtered['v'].min(), df_filtered['v'].max(), 100)
    yi = np.linspace(df_filtered['bs'].min(), df_filtered['bs'].max(), 100)
    xi, yi = np.meshgrid(xi, yi)

    # Interpolate alpha* and gamma* values for the grid
    zia = griddata((df_filtered['v'].dropna(), df_filtered['bs'].dropna()),␣
↪df_filtered['alpha*'].dropna(), (xi, yi), method='cubic')
    zig = griddata((df_filtered['v'].dropna(), df_filtered['bs'].dropna()),␣
↪df_filtered['gamma*'].dropna(), (xi, yi), method='cubic')

    # Apply masks based on various conditions to handle invalid or extreme data␣
↪points
    mask1 = griddata((df_filtered['v'], df_filtered['bs']), df_filtered['bI'] <␣
↪0, (xi, yi), method='nearest')
    mask2 = griddata((df_filtered['v'], df_filtered['bs']),␣
↪df_filtered['I_bar'] < 1e-3, (xi, yi), method='nearest')
    mask3 = griddata((df_filtered['v'], df_filtered['bs']),␣
↪df_filtered['E_bar'] < 1e-3, (xi, yi), method='nearest')
    mask_combined = mask2 | mask3

    # Set invalid data points to NaN for alpha* and gamma*
    zia[mask1] = np.nan
    zig[mask1] = np.nan

    # Plot alpha* values on a 3D surface plot
    ax1 = fig.add_subplot(2, len(n_values), i, projection='3d')
```

```
    surf_alpha_1 = ax1.plot_surface(xi, yi, zia, cmap='viridis', edgecolor='none')
    ax1.plot_surface(xi, yi, np.where(mask_combined, zia, np.nan), cmap='Greys', edgecolor='none', zorder=1)
    ax1.plot_wireframe(xi, yi, np.where(mask_combined, zia, np.nan), color='black', alpha=0.7)
    ax1.set_xlabel(r'$(v)$', fontsize=50)
    ax1.set_ylabel(r'$(bs)$', fontsize=50)
    ax1.set_title(f'n = {n}', fontsize=60)
    ax1.view_init(elev=45)

    # Plot gamma* values on a 3D surface plot
    ax2 = fig.add_subplot(2, len(n_values), len(n_values) + i, projection='3d')
    surf_gamma_1 = ax2.plot_surface(xi, yi, zig, cmap='inferno', edgecolor='none')
    ax2.plot_surface(xi, yi, np.where(mask_combined, zig, np.nan), cmap='Greys', edgecolor='none', zorder=1)
    ax2.plot_wireframe(xi, yi, np.where(mask_combined, zig, np.nan), color='black', alpha=0.7)
    ax2.set_xlabel(r'$(v)$', fontsize=50)
    ax2.set_ylabel(r'$(bs)$', fontsize=50)
    ax2.view_init(elev=45)

# Adjust layout to ensure clear visibility
plt.tight_layout()

# Display the plot
plt.show()
```
